# Transfer of Avatar Training Effects to Investigative Field Interviews of Children Conducted by Police Officers

**DOI:** 10.3389/fpsyg.2022.753111

**Published:** 2022-07-06

**Authors:** Kristjan Kask, Francesco Pompedda, Annegrete Palu, Karolyn Schiff, Mari-Liis Mägi, Pekka Santtila

**Affiliations:** ^1^Laboratory of Experimental Psychology, School of Natural Sciences and Health, Tallinn University, Tallinn, Estonia; ^2^School of Natural and Social Sciences, University of Gloucestershire, Cheltenham, United Kingdom; ^3^Institute of Psychology, University of Tartu, Tartu, Estonia; ^4^Viljandi Hospital, Viljandi, Estonia; ^5^Faculty of Arts and Sciences, New York University Shanghai, Shanghai, China; ^6^NYU-ECNU Institute for Social Development, New York University Shanghai, Shanghai, China

**Keywords:** child sexual abuse (CSA), training with virtual reality, interview training, investigative interviewing, serious games

## Abstract

Previous research with students and some professional groups (psychologists) has demonstrated that repeated feedback in simulated investigative interviews with computerized child avatars improves the quality of interviews conducted with real children who have witnessed a mock event. However, it is not known whether this type of training would improve the quality of investigative interviews with actual child victims and witnesses of physical and sexual abuse. Twenty-two police investigators participated in the study. Half of them received feedback during four simulated interviews whereas the other half received no feedback during four such interviews followed by another four interviews after which they also received feedback. Transcripts of interviews both before and after the training were coded for interview quality. Receiving feedback after the simulated interviews increased the proportion of recommended questions both within the simulations and, importantly, also during interviewing with actual child victims and witnesses. This study demonstrated for the first time transfer of learning from simulated interviews to actual investigative interviews.

## Introduction

It is well known that interviewing children in alleged child sexual abuse (CSA) cases is a multi-faceted and a challenging task (Powell et al., 2008). To master these skills, specialized training is needed (Benson and Powell, 2015). There are several best-practice guidelines that outline how these interviews should be conducted (Lyon, 2014; Lamb et al., 2018) but several studies have shown that interviewers have difficulty complying with these instructions in practice (see Lamb, 2016). Suggesting that the problem can be solved, simulated interviews with child avatars combined with feedback have repeatedly been shown to increase the proportion of recommended [i.e., invitations (‘Tell me what happened’) and open-ended (‘Where were you standing?’) questions within the avatar training itself (Pompedda et al., 2015, 2017, 2021; Krause et al., 2017; Pompedda, 2018; Haginoya et al., 2020)]. Also, these positive training effects transfer, at least to some extent, to interviews with children who have experienced a mock event (Pompedda et al., 2021). However, whether the training effects transfer to interviews with actual child victims or witnesses of abuse has not yet been addressed.

It is known that open-ended questions, such as invitations, are more likely to elicit accurate accounts from children (Lamb et al., 2018). Despite empirical evidence being clear about what kinds of questions should be used, previous research has shown that not recommended questions are frequently used in investigative interviews such as option-posing (‘Did he push you once or twice?’), repeated (repeating a question in the exact same wording) or suggestive questions (‘You were there, weren’t you?’, Lamb et al., 2018). It seems that transferring theoretical knowledge of best-practice guidelines into real life interviews is surprisingly difficult (Sternberg et al., 2001). Even after intensive training including practical exercises and feedback, the quality of investigative interviews is slow to change (Johnson et al., 2015). For example, Powell et al. (2016) demonstrated that the overall proportion of open-ended questions after an intensive training program was still low, and even if an improvement was present, it decreased quickly after the end of the training (Price and Roberts, 2011).

Even an interview where a child gives a narrative account of sexual abuse that is substantiated by corroborative evidence concerning its central details may include an unknown number of erroneous details resulting from the use of suggestive questions. To overcome this problem, serious games combined with feedback has been proposed as a solution to improve the quality of CSA interviews (Benson and Powell, 2015; Powell et al., 2016). In previous experiments with students (Pompedda et al., 2015, 2017, 2021; Krause et al., 2017; Pompedda, 2018; Haginoya et al., 2020) and psychologists (Pompedda et al., 2021), interview quality in simulated investigative interviews with avatars was considerably improved in just 1 h when the interviewers were given feedback on their performance after each interview. Pompedda et al. (2021) showed the transfer effect, namely that those interviewers who were provided feedback after each avatar interview, asked more recommended questions from children. However, in the two experiments included in Pompedda et al. (2021), the children had experienced a non-abuse mock event and it is therefore not certain that similar transfer would be demonstrated in field interviews with actual abuse victims.

There are a limited number of studies of transfer effects using other training protocols. Powell et al. (2016) provided the trainees 36 h of computer-based activities over several months that involved immediate feedback regarding interviewing performance. The effect of training on mock interviews with actors was then examined. After the training 75% of the questions asked by the participants were open-ended questions, and this effect lasted 3 and 6 months later. In a similar study by Benson and Powell (2015) the proportion of open questions increased from 30% pre-training to 58% post-training, and the improvements were still present 12 months later. In a study by Brubacher et al. (2015), teachers interviewed a 5-year-old avatar three times over a week while getting immediate feedback on their performance. One week later they interviewed a trained research assistant who portrayed a child. After the training the prevalence of open questions was 50% compared to 13% pre-training. These results show promising transfer effects from training approaches that are similar to the one examined in this study.

As investigative interviewing is cognitively demanding, serious games is ideal to increase training effect transfer in the light of theoretical frameworks for understanding how these effects can be facilitated (e.g., Blume et al., 2010). Cognitive Load Theory (Ayres and Paas, 2009) suggests that transfer can be facilitated by minimizing cognitive load due to external factors (i.e., instructional material used to present the content). This will allow the learner to devote more cognitive resources to the actual learning, that is, germane cognitive load (Mugford et al., 2013). Effective learning of a complex task such as investigative interviewing requires the automation of schemata that reduces the load on working memory during task performance (Paas et al., 2003). Avatar training as an active learning (practice) approach should ensure schemata automatization (Sweller et al., 1998). Avatar training setup has been planned with this in mind (see Pompedda, 2018). Instead of reading in a guideline or being explained by an expert, the participants learn about the negative effects of suggestive interviewing through their questioning and the feedback they are provided. The avatar training task is complex and consists of mixed practice achieved by several methods. First, the avatars have different memory contents (abuse vs. no abuse scenarios). Second, the avatars’ responses are driven by probabilistic algorithms resulting in response patterns between the interviews varying considerably. Finally, the avatars are of different ages and abuse and non-abuse cases follow each other randomly. Transfer is more likely to be present in occasions that have a close resemblance to the training situation, both regarding structural and surface similarity (e.g., Soveri et al., 2017). In the context of training with avatars, structural similarity refers to the avatars having response algorithms that mimic those of real children of a specific age. This means that the actual central task of eliciting information shares its main cognitive features between the training and the transfer situations. Surface similarity refers to the fact that the avatars look and talk like actual children. In this way, when the interviewers later interview actual children, the way they look and talk will facilitate recall of task relevant information.

### Current Study

Our aim was to examine whether avatar training coupled with feedback would transfer into investigative interviews with children in actual criminal cases of child sexual and physical abuse conducted by Estonian police officers. The police officers who participated in the study were provided with avatar training with feedback or without feedback on their performance. Investigators provided the researchers a transcript of an interview with a real child victim or witness prior the avatar training and after avatar trainings. If the participants did not conduct a field interview with a child within a month after avatar training, they participated in a refresher session approximately every month while waiting to conduct one or more field interviews.

We tested the following hypotheses:

(1) Effect of feedback manipulation in simulated interviews with avatars: we expected that receiving feedback during the avatar training would result in the interviewers using a higher proportion of recommended questions in the avatar training compared to the group that did not receive feedback.

(2) Transfer of the effect of the feedback manipulation in avatar interviews into interviews with children in actual child sexual or physical abuse cases: we expected the improvements from receiving feedback during avatar training to transfer into actual investigative interviews with children so that interviews conducted after (vs. before) the training coupled with feedback would have a higher proportion of recommended questions.

Anecdotal evidence suggests that interviewers sometimes justify the use of not recommended questions and interview practices by the difficulties they feel children have in responding to open ended questions. For this reason, we also explored whether there were any differences in the likelihood of the children revealing abuse in interviews conducted before and after the training.

## Materials and Methods

### Participants

A total of 22 police investigators took part in the study. Two participants dropped out after the first four interviews without feedback and did not receive training with feedback; three investigators did not provide any field interview transcripts. Thus, the number of final sample is 17 as at least one field interview was provided by 17 investigators (age *M* = 40.5, *SD* = 7.6, range 25–49, 2 men). Three were native Russian speakers and 14 native Estonian speakers. Although the official language of criminal proceedings in the Republic of Estonia is Estonian, investigative interviews may take place in other languages. On average, the investigators had worked within the police for 14.3 (*SD* = 7.9) years and had conducted 40.2 (*SD* = 40.4) videotaped interviews with children in general and 15.6 (*SD* = 23.70) videotaped interviews in CSA cases. During the last year, the average number of videotaped interviews with children was 11.4 (*SD* = 11.2) and of videotaped interviews in CSA cases 4.4 (*SD* = 7.7). The investigators did not receive any reward for their participation.

#### Field Interview Related Variables

Of the actual child interviewees (*N* = 36) in the field interviews, 61% were boys. On average, the interviewees were 8.3 (*SD* = 2.6, range 3.5–12.9) years old, half (49%) were 8 years or younger. Most (86%) were Estonian speakers, the rest Russian speakers and all were interviewed in their mother tongue. A minority (32%) of the interviews involved CSA allegations, the rest involved physical abuse investigations. The interviews lasted on average for 30.3 (*SD* = 19.9) minutes. A majority (91%) of the interviews were the first interview regarding the allegation in question. Also, in a majority (79%) of the interviews, the child confirmed the abuse allegation (44% had done so before the interview as well according to hear-say evidence). On average, 126.4 (*SD* = 290.2, *Md* = 14, range 1–1,460) days had passed since the last suspected criminal incident.

### Study Design

**FIGURE 1:**
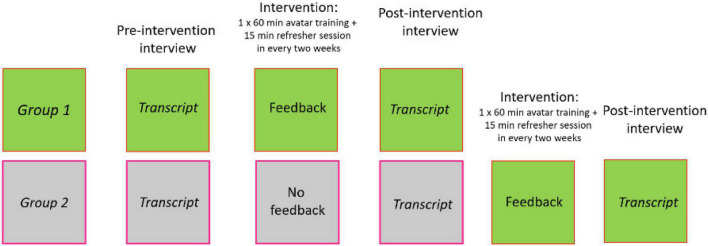
Experimental design.

The participants were first matched into pairs based on their background variables concerning gender, age, how long the participants had been working in the field, how many years of videotaped interviews they had conducted, both in CSA cases and in general, total number of videotaped interviews, total number of videotaped interviews in the last year, and interviews conducted in Russian or Estonian. After this they were randomly assigned to either the Feedback (*n* = 11), or the combination of No-Feedback/Feedback (*n* = 11) group. There were no significant differences between the two groups on any of these variables. Participants were asked to provide a transcript of investigative interview from investigation of child sexual or physical abuse before the avatar training and after each avatar training session. The study design is outlined in Figure 1.

In Feedback group, the investigators participated in one avatar interview training session where they conducted four avatar interviews and were given feedback on their performance after each interview. Each participant in this group was also asked to provide two transcripts of investigative interviews from investigations of child sexual or physical abuse: (a) one before the avatar training, and (b) one after the training.

In No Feedback/Feedback group, the investigators participated in two avatar interview training sessions – in the first session they interviewed four avatars and did not receive feedback on their performance and in the second session they also interviewed four avatars but now received feedback on their performance. Each participant in this group was asked to provide three transcripts of investigative interviews again from investigations of child sexual or physical abuse: (a) one before the avatar training, (b) one between the two avatar training sessions, and (c) one after the second avatar training. Two investigators decided to withdraw participation in the study after completing the first avatar training session, resulting in the final number of participants in this group being nine.

### Materials

#### Simulated Investigative Interviews Using EIT

We performed simulated interviews with avatars using the same procedure as in previous studies (see Pompedda, 2018; Pompedda et al., 2021, for a detailed description). Operators who had been previously trained in the use of the software and coding scheme, listened to each question asked by the interviewer, categorized it (e.g., as option-posing) after which a response algorithm in the software was activated. The software then launched an appropriate video clip containing the avatar’s response. The training tool consists of 16 different avatars (computerized children) with different scenarios to be investigated. Half of the avatars are emotional (i.e., crying) and half are neutral. Half of the stories include abuse while the other half do not, and half of the avatars are 4 years old while the other half are 6 years old. The avatars have predefined memories and details of the alleged CSA scenarios. The avatars respond to the interviewers’ questions providing predefined details through probabilistic response algorithms that are derived from studies on children’s responses to different question types in experimental studies where the accuracy of such responses can be ascertained. These algorithms increase the ecological validity compared to other training tools as they provide the interviewers with realistic response patterns.

#### Field Interviews

The investigators were asked to provide two (Feedback group) or three (No Feedback/Feedback group) transcripts of investigative interviews with children from cases in which sexual or physical abuse was suspected (see Table 1 for the numbers of interviews from the different phases of the experiment). The investigators could choose themselves which transcript to provide. The transcripts were anonymized by a representative of the Estonian Police and Border Guard Board to withdraw all sensitive and identifiable information about the case.

**TABLE 1:** Field interviews conducted before and after avatar training with feedback.

	Interview		

	Before avatar training	After no feedback training	After feedback training
Group 1	9^a^		8^b^
Group 2	8^a^	7^a^	3^b^

*Shaded numbers indicate interviews conducted before avatar training with feedback. In subsequent statistical analyses of the transfer effect, the interviews with suprascript^a^ were compared to interviews with suprascript^b^.*

#### Coding of Interviews

Both the interviews with avatars and the interviews with the children were coded for question types and the details elicited from the avatar/child. For coding question types in avatar and child interviews, and the details recalled from the avatar interviews, we used a scheme used in previous studies (e.g., Pompedda et al., 2021). One detail was defined as a narrative phrase that the avatar provides in response to a recommended question (e.g., I was playing soccer with my uncle). In avatar interviews, the details answered by avatars in response to interviewers’ questions were coded as correct or incorrect. As in interviews with real children we could not code the veracity of the details, we coded children’s answers as following: unintelligible answers (impossible to tell, such as ‘I was doing/—-/), narrative details (‘I heard him coming to the room’), non-verbal answers (yes/no; don’t know; other), yes/no answers, don’t know answers, don’t remember answers, don’t understand answers, correcting the interviewer (‘No, I said two’), change in arguments (‘Actually, it was…’), asking a question instead of answering (‘What was your name again?’), and not answering at all. We also calculated shifts in children’s answers (if the child changed his/her answers in response to questions).

#### Inter-Rater Reliability

To investigate the reliability of the coding of the field interviews, one interview with a total of 108 questions was coded by two coders (KS and MLM). We found adequate levels for recommended and non-recommended questions (percentage agreement 85%, Cohen’s κ = 0.704 [0.570–0.832], *p* < 0.001), question type (percentage agreement 89% Fleiss’ κ = 793 [0.692–0.894], *p* < 0.001) and answer type (percentage agreement 86% Fleiss’ κ = 0.774 [0.644–0.903], *p* < 0.001). The inter-rater reliability was lower for number of details (percentage agreement 84% Kendall’s tau τb = 0.571, *p* < 0.01) which could be due to difficulties in discriminating details (nouns, verbs) from grammar (cases) in Estonian language.

### Procedure

All investigators who were recruited identified a videotaped child interview from before the start of the research project. Investigators in Feedback group were provided with Avatar training combined with feedback after which one or two subsequent interviews were obtained from them. In Feedback group, nine interviews were obtained from before the training, eight interviews immediately after training and one more subsequent interview. Investigators in No Feedback/Feedback group were first provided with Avatar training without feedback after which one additional interview was obtained from them. This was followed by Avatar training with feedback after which attempts were made to obtain an additional interview from them. In No Feedback/Feedback group, eight interviews were obtained from before the first training, seven from after the first training and three from after the second training.

#### Training With Avatars

The experiment assistant met the participants individually in their work environment. They received minimal instructions about best practices in child interviewing, which consisted of information on the question types to use and to avoid during interviews with children. In this phase, we asked two questions to check if the participants had understood the provided guidelines. For example, ‘If the child doesn’t provide any detail regarding the alleged situations of abuse, the interviewer should ask questions related to the alleged situation, such as: Did your football coach touch you?’ In this case, the correct answer is ‘No.’

For each participant, four out of sixteen possible avatars were randomly selected for the training session. The selection of the avatars was randomized while making sure that each interviewer had avatars of different age (4/6 years old), gender (male/female) and scenarios (abuse/no-abuse). Hence, each participant received two abuse and two no abuse situations. The order of the avatars was randomized. Because it was impossible to balance age by abuse situation across the four training interviews per participant (within four avatars), this was instead balanced within groups.

Next the participants were given oral instructions on the different phases of the study after which they were given the background scenario of the first avatar to be interviewed. Each interview lasted a maximum of 10 min and, at the end of each interview, participants were asked to explain what they thought had happened with as many details as they could. However, they were also informed that ‘I do not have enough information to draw a conclusion’ was an acceptable answer. After each interview, participants in the feedback group received a combination of outcome feedback (information about what had actually ‘happened’ to the avatar) and process feedback, which consisted of examples of four questions they used during the interview (e.g., ‘Do you play with your daddy?’), and a description of the question type category these belonged to (e.g., option posing) and why this category should or should not be used. Also, a reflection task was used where the participant was asked to transform used inappropriate types of questions into appropriate types of questions, for example, multiple questions to open questions.

In total, participants received feedback on four questions (two recommended and two non-recommended questions) after each interview. Feedback was provided in a way that covered as many question types as possible: New question types used by the interviewer were prioritized during the feedback session while always providing feedback for two recommended and two non-recommended questions. The avatar interview session lasted for about 90 min. At the end of the training session with the avatars, all participants were provided with advice on how to conduct interviews with children, and instructions extracted from the NICHD protocol (Lamb et al., 2007).

#### Refresher Sessions

In case the investigator did not have any field interview with a child in a sexual or physical abuse case within 1 month, they participated in a refresher session. The refresher sessions were included to maintain (or re-establish) the training effect. A refresher session consisted of two interviews with new avatars. If the previous training was conducted without feedback, then the refresher session was also conducted without feedback. If the previous training was with feedback, participants received feedback after both refresher session interviews. The refresher session lasted on average 30 min.

### Statistical Analyses

For the simulated avatar interviews, we first used a Mixed ANOVA to test if there were differences between Feedback and No Feedback/Feedback groups in the first four avatar interviews both groups conducted. As described above, Feedback group received feedback after each of these four interviews whereas No Feedback/Feedback did not receive any feedback. Second, we also tested if No Feedback/Feedback group, that conducted four interviews without feedback and then four interviews with feedback, increased use of recommended questions during the latter set of interviews. For the field interviews, the original analytical plan was to first test whether the interviews improved in Feedback group after Avatar training with feedback but not in No Feedback/Feedback group after Avatar training without feedback and whether the second interviews were of better quality in Feedback group. Finally, we wanted to test for improvement in interview quality from second to third interview in No Feedback/Feedback group given that, in between these, Avatar training with feedback was provided. However, due to lower than expected numbers of investigators recruited, and high drop-out rates in No Feedback/Feedback group between the second and third interviews, we decided to simplify the analytical plan to increase statistical power: we compared all interviews that were conducted before Avatar training with feedback with all interviews that were conducted after Avatar training with feedback (see Table 1). This ignores some dependencies in the data. For all comparisons, we used one-sided Mann–Whitney *U-*tests with exact significance testing.

### Ethics

The board of research ethics at Åbo Akademi University (Finland) and Tallinn Medical Research Ethics Committee (Estonia) approved the study before the data collections commenced. The study was also approved by the Police and Border Guard Board of Estonia and Office of the Prosecutor General of Estonia. The study was conducted following the guidelines of the Declaration of Helsinki.

## Results

### Avatar Training Interviews

#### Correlations Between Proportion of Recommended Questions and Other Interview Quality Indicators in the Avatar Interviews

Table 2 shows the correlations between the proportion of recommended questions and the other indicators of interview quality in the avatar interviews, that is, correct details, wrong details, the proportion of correct details out of all details as well as the number of correct conclusions. All correlations were in the expected direction. Asking more recommended questions resulted in significantly more correct details being elicited from the avatar and significantly more correct conclusions being reached, and in significantly less wrong details being elicited from the avatar. These results show that the avatar training simulation worked as intended.

**TABLE 2:** Pearson correlations between proportion of recommended questions and correct and wrong details being elicited from the avatar as well as number of correct conclusions reached (*n* = 172).

	Proportion recommended questions	Incorrect details	Correct details	% correct details
Incorrect details	−0.613**			
Correct details	0.572**	−0.177*		
% correct details	0.706**	−0.699**	0.600**	
Correct conclusion	0.236**	−0.153*	0.269**	0.237**

Correct details only include correct details related to the suspected abuse. **Correlation is significant at the 0.01 level (2-tailed); *correlation is significant at the 0.05 level (2-tailed).

#### The Effects of Feedback on the Proportion of Recommended Questions in the Avatar Interviews

**FIGURE 2:**
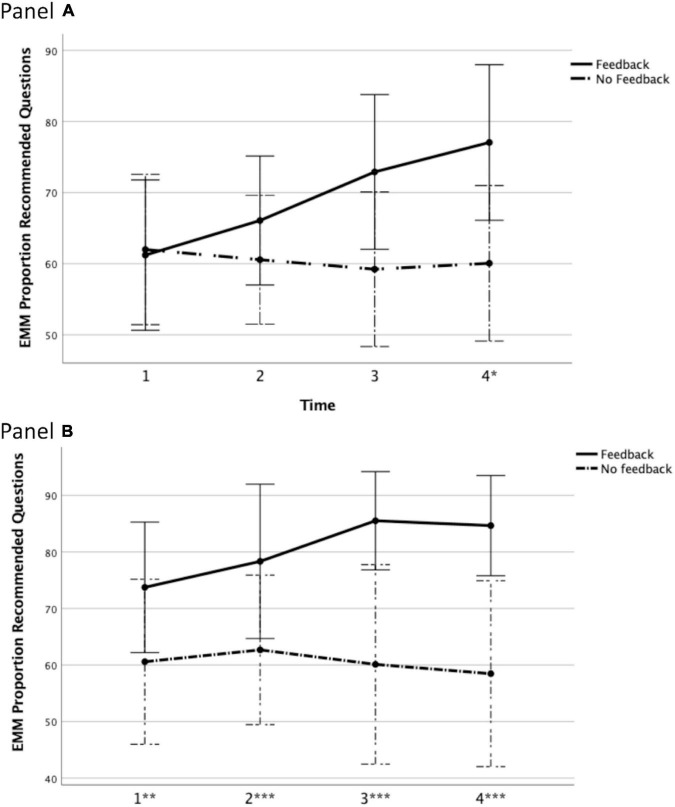
Effects of feedback on proportion of recommended questions during the avatar training interviews. **(A)** Estimated marginal means (EMM) of the difference in initial four interviews between Group A (*n* = 11; received feedback) and Group B (*n* = 11; did not receive feedback). **(B)** Difference in Group B’s initial four interviews (*n* = 9; did not receive feedback) and subsequent four interviews (*n* = 9; received feedback). *Significant pairwise difference between conditions at the indicated interview. Error bars show 95% confidence intervals. Bonferroni correction applied *p < 0.05, **p < 0.01, ***p < 0.001 (2-tailed).

First, we tested if there were differences between Feedback group (*n* = 11) and No Feedback/Feedback group (*n* = 11) in the first four avatar interviews both groups conducted. As described above, Feedback received feedback after each of these four interviews whereas No Feedback/Feedback group did not receive any feedback. We expected Feedback group to increase the proportion of recommended questions used whereas No Feedback/Feedback group was expected to show no improvement. A Mixed ANOVA with Feedback (Between-Subjects) and Time (Within-Subjects) as independent variables showed that there was no significant main effect of Group, *F*(1,20) = 1.91, *p* = 0.182, $\eta_{\text{p}}^{2}$ = 0.09, 1 – β = 0.26, but that there was a significant main effect of Time, *F*(3,60) = 3.22, *p* = 0.029, $\eta_{\text{p}}^{2}$ = 0.14, 1 – β = 0.71. Importantly, and supporting our hypothesis, the interaction between Group and Time was significant, *F*(3,60) = 5.54, *p* = 0.002, $\eta_{\text{p}}^{2}$ = 0.22, 1 – β = 0.93. Figure 2A shows that, as expected, Feedback group increased the proportion of recommended questions they used whereas the proportion remained relatively stable in No Feedback/Feedback group even if the confidence intervals overlap for the pair-wise comparisons.

Second, we also tested if No Feedback/Feedback group, that conducted four interviews without feedback and then four interviews with feedback, increased their use of recommended questions during the latter set of interviews. Two participants completed only the first four interviews without the feedback and they have been excluded from the analysis that in the end included nine participants. A Within-Subjects ANOVA with Feedback and Time as independent variables showed that receiving feedback during the interviews compared to not receiving feedback had a statistically significant effect, *F*(1,8) = 55.63, *p* < 0.001, $\eta_{\text{p}}^{2}$ = 0.87, 1 – β > 0.99, whereas the main effect of time was not significant, *F*(3,24) = 2.60, *p* = 0.076, $\eta_{\text{p}}^{2}$ = 0.24, 1 – β = 0.56. Importantly, and again supporting our hypothesis, the interaction between receiving Feedback and Time was significant *F*(3,24) = 3.85, *p* = 0.022, $\eta_{\text{p}}^{2}$ = 0.32, 1 – β = 0.75. Figure 2B shows that the proportion of recommended questions increased when feedback was provided whereas it remained stable when no feedback was provided. During the last interview, the confidence intervals do not overlap between the conditions.

### Transfer to Field Interviews

#### Randomization Check

We compared interviews conducted before avatar training with feedback to interviews conducted after avatar training with feedback in terms of the interviewer experience variables as well as child age and days that had passed since the last suspected criminal incident. There were no differences (all *p*s > 0.178, 2-tailed exact Mann–Whitney *U*-tests). Also, there was no relationship between the experimental condition of the interview and whether the child had reportedly talked about the alleged incident already prior to the interview nor if the alleged incident contained a CSA allegation or not (both *p*s > 0.434, 2-tailed exact Chi-Square tests).

#### Interview Quality

On average, the interviewers used 82.4 (*SD* = 95.0) recommended and 64.3 (*SD* = 50.3) not recommended questions. The average proportion of recommended questions was 0.52 (*SD* = 0.15). There were an average of 32.8 (*SD* = 49.9) facilitators, 1.4 (*SD* = 3.0) broad invitations, 6.2 (*SD* = 6.1) narrow invitations, 41.2 (*SD* = 46.9) directives and 0.8 (*SD* = 1.6) requests for clarification. There were an average of 35.8 (*SD* = 28.7) option-posing, 4.8 (*SD* = 6.5) suggestive, 1.5 (*SD* = 2.9) unspecific suggestive, 5.1 (*SD* = 8.5) multiple, and 11.9 (*SD* = 11.5) long/confusing questions as well as 1.2 (*SD* = 3.3) repetitions and 4.1 (*SD* = 4.6) questions related to time, feelings and imagination. In terms of the child responses, there were 87.7 (*SD* = 88.6) narrative answers and 6.6 (*SD* = 10.2) don’t remember/don’t know/don’t understand/correct interviewer type of answers as well as 3.7 (*SD* = 5.9) unintelligible answers and 41.1 (*SD* = 50.1) yes/no answers. The children changed their answers on average 1.1 (*SD* = 1.6) times and the total number of details was 892.5 (*SD* = 961.1).

#### Differences in Interview Quality Between Interviews Conducted Before and After Avatar Training With Feedback

Table 3 shows the results for the individual question types as well as for the other interview quality indicators. Even though the number of recommended and not recommended questions did not differ significantly between the two types of interviews, the proportion of recommended questions increased significantly from 0.48 (*SD* = *0.12*) to 0.59 (*SD* = *0.18*), *p* < 0.022. The effect is due to a decrease in not recommended questions.

**TABLE 3:** Differences in interview quality between interviews conducted before and after avatar interview training combined with feedback.

	Before		After		Mann–Whitney *U*	Exact sig. (1-tailed)
	*M*	*SD*	*M*	*SD*		
Recommended questions	82.5	107.6	82.0	67.3	117.0	0.187
Not recommended questions	70.6	55.5	51.7	36.5	118.5	0.201
Proportion of recommended questions	0.5	0.1	0.6	0.2	84.0	0.022
Facilitators	36.8	58.2	24.8	27.2	132.5	0.355
Broad invitations	1.1	2.9	2.0	3.3	97.0	0.046
Narrow invitations	6.2	6.7	6.3	5.0	125.5	0.271
Direct questions	37.6	46.2	48.5	49.4	106.0	0.104
Clarifications	1.0	1.9	0.4	0.8	125.5	0.265
Option-posing questions	39.2	32.2	28.8	19.5	123.0	0.246
Suggestive questions	6.2	7.3	1.8	3.4	86.0	0.023
Unspecific suggestive questions	2.1	3.4	0.3	0.5	90.0	0.025
Multiple questions	6.1	10.0	3.1	3.9	116.5	0.180
Long and/or confusing questions	11.9	11.9	11.9	11.1	142.5	0.484
Repetitions	1.5	3.9	0.7	1.0	143.0	0.499
Questions related to time, feelings and imagination	3.7	4.2	5.1	5.4	110.5	0.133
Unintelligible	4.7	6.5	1.8	4.2	87.5	0.035
Narrative detail answers	83.4	97.1	95.9	73.0	99.0	0.090
Yes or no answers	51.3	59.0	21.4	12.2	80.5	0.023
Don’ t remember/know/understand/correct answers	7.0	11.9	5.8	5.9	125.5	0.336
Child changes the response	1.4	1.8	0.4	0.7	99.5	0.077
Number of details	882.3	1101.3	912.8	634.5	122.0	0.238

In the interviews conducted after avatar training with feedback significantly more broad invitations (82% increase), less suggestive (71% decrease), and less unspecific suggestive questions (86% decrease) were used. Also, child responses were significantly less likely to be unintelligible (62% decrease) or be yes/no responses (58% decrease). Finally, there was no significant difference in the likelihood of children who had not confirmed the criminal incident before to do so in the interview between the two interview groups, that is, the decreased use of more direct questions in the interviews conducted after the feedback training did not make it less likely for the children to reveal that they had been abused.

## Discussion

In the present study we examined the effects of simulated Avatar interviews coupled with feedback in a group of Estonian police investigators. Confirming the results of previous research with student or practitioner (psychologists) samples, we demonstrated that feedback enhances the use of recommended questions within a simulated avatar training itself. We also showed that actual field interviews in child abuse investigations conducted after training paired with feedback have a better quality on multiple indicators compared to interviews conducted before such training. This is the first time transfer has been shown in actual field interviews.

### Avatar Training Effects

Compared to previous studies with both student and professional samples (see Pompedda, 2018), Estonian police officers used more recommended questions in the Avatar interviews to start with. In all conditions, the starting levels were over 60%. This suggests that previous training efforts in Estonia have been effective in enhancing the quality of investigative interviews. Currently, if an investigator specializes in interviewing minor victims and witnesses, they will have the opportunity to participate in a 2–3 weeks long intensive course where first the theoretical background of investigative interviewing with minors is covered based on the NICHD protocol (Lamb et al., 2018). This is followed by a feedback assisted practice session where the investigator has to interview one of their colleagues portraying to be a child victim or witness of physical or sexual abuse. The results suggest that this approach is at least to some extent effective in improving interview quality given that previous studies have suggested a surprisingly stable (low) quality irrespective of context.

Clear differences between the participants receiving (vs. not) feedback started to emerge at the fourth avatar training interview. This suggests that adding one or two simulated interviews to the training phase might be preferable to maximize transfer. Also, it is evident in the analysis within the group that received delayed feedback (No Feedback/Feedback group) that these participants started showing improvements immediately after receiving feedback compared to the previous four interviews where they received no feedback. This suggests that they learned some of the general task demands of the simulation (unrelated to question types) already during the phase without feedback allowing them to take in the feedback with less distractions during the feedback phase. Thus, a solution that could be tested in the future would be to begin with one or two avatar interviews without feedback for investigators to familiarize themselves with the software and the procedure before moving to avatar interview with feedback which could maximize the effect that feedback has on learning.

Even though the starting level was better than in previous research, simulated avatar interviews with feedback resulted in significant increases in the use of recommended questions in both the between and the within-subject comparisons. This is important to note despite the small sample size. Thus, we can say that our results support Hypothesis 1 (we expected that receiving feedback during the avatar training would result in the interviewers using a higher proportion of recommended questions in the avatar training compared to the group that did not receive feedback). In addition, the improvements in the use of recommended questions were negatively correlated with the number of incorrect details and positively correlated with the number of correct details elicited from the avatars as well as the likelihood of the interviewer drawing the correct conclusion about what had ‘happened’ to the avatar. This is also in line with previous research (e.g., Pompedda et al., 2015).

### Transfer Effects

We found support for a transfer effect so that the quality of the real interviews with child victims and witnesses of sexual and physical abuse improved (operationalized as the increase in the proportion of recommended questions) after simulated avatar interviews paired with feedback. Thus, our results support Hypothesis 2. The effect was present despite some limitations. The small sample size limited the statistical power of the analyses. Also, the field interviews were quite heterogeneous in terms of type of abuse, time since the last suspected event, and child age. These factors can be expected to limit the likelihood of successful transfer even though the training setup used had been created to provide variable practice. We have to bear in mind as well that our training included minimal theoretical components suggesting again that such components may not be necessary to achieve transfer of a complex skill such as interviewing. This is not surprising given the theoretical models of transfer.

On average, the proportion of recommended questions asked by the investigators before the avatar training was 0.52 which is comparable to other similar studies (see Benson, 2015). The number of recommended questions did not differ in interviews before and after the avatar training, however, the number of not recommended questions decreased, although not significantly. Still, the interviewers used a large proportion of directive questions in their interviews with real children. It is known from the Estonian investigators’ interviewing quality from interviewing conducted between 2004 and 2008 (Kask, 2012) that the proportion of invitations was low (4.5% of the questions) whereas the main question types asked were direct (40%), option-posing (34%), and suggestive questions (5%). It could be that when investigators aim to improve their interviewing skills then it is difficult to change their style rapidly from closed questions to invitations and therefore find it easier to use directive questions instead. In the current study, the decrease in option-posing and suggestive questions led to an increase of directive questions. Thus, interviewers transformed several suggestive and option-posing questions into question types that are more appropriate in an investigative context. It is of question how to make the next step in increasing the quality of the interviews by decreasing the number of directive questions and increasing the number of invitations. Finally, the likelihood of disclosing was not affected by the training which is a potential worry as it is difficult to disclose abuse. As in the field setting the ground truth is not known, thus disclosing cannot be interpreted unambiguously.

### Strengths and Limitations

First, as already mentioned above, the sample size of the current study was small. As the number of investigators investigating crimes against minors in Estonia is classified, we cannot estimate which proportion of the investigators’ population participated in our study. Therefore, we have to be careful in making generalizations based on the results of our study. Future research should replicate our results in a larger sample of investigators.

However, due to small sample size it was possible for us to get necessary permissions to analyze anonymized interviews still under proceedings. This is rare as usually the permission to get acquainted with the interview protocols is made available to the researchers when the verdict is final (which, in cases, mean that the case has been processed in all three levels of the court system, i.e., county, circuit, and supreme courts).

It is also important to add elements to the avatar training to expand the coverage of interview skills. For example, the revised protocol of NICHD emphasizes supportive interviewing (Hershkowitz et al., 2017) as the children who are victims of violent crimes may be still reluctant to disclose the details of what happened to the investigators. Future studies should therefore focus more on comprehensive interview skills such as support.

It is worthwhile to note that not all closed questions decrease the quality of interviews. After recommended questions, appropriate closed questions, based also on the age of the interviewee, and utilized at the end of the interview, may help the witnesses to provide more information about what happened which can be useful in legal decision-making (see Verkampt et al., 2019 but also Griffiths and Milne, 2006; Walsh and Bull, 2015). Therefore, in future studies it is important to develop training to learn the use of appropriate closed questions as well.

## Conclusion

The results of the present study expand our knowledge about the efficacy of simulated training. The transfer effect of knowledge to field practice was demonstrated first time in a sample of police officers. The quality of the interviews while providing feedback to the avatar training increased the proportion of recommended questions both in avatar interviews as well as in investigative interviews with real child victims and witnesses of sexual and physical abuse. The effectiveness of avatar training has now been validated in various cultural contexts such as Finland (Krause et al., 2017), Italy (Pompedda et al., 2015, 2017), Estonia (Pompedda et al., 2021), and Japan (Haginoya et al., 2020, 2021).

## Data Availability Statement

The raw data supporting the conclusions of this article will be made available by the authors, without undue reservation, to any qualified researcher.

## Ethics Statement

The studies involving human participants were reviewed and approved by Åbo Akademi University (Finland) and Tallinn Medical Research Ethics Committee (Estonia). The patients/participants provided their written informed consent to participate in this study.

## Author Contributions

KK, FP, and PS designed the study, drafted the Methods section of the manuscript, and wrote the manuscript. FP created the materials and mock events scripts. AP, KS, and M-LM performed the experiments. FP and PS analyzed and interpreted the data. AP critically reviewed the manuscript. All authors listed have made a substantial, direct, and intellectual contribution to the work, and approved it for publication.

## Conflict of Interest

The authors declare that the research was conducted in the absence of any commercial or financial relationships that could be construed as a potential conflict of interest.

## Publisher’s Note

All claims expressed in this article are solely those of the authors and do not necessarily represent those of their affiliated organizations, or those of the publisher, the editors and the reviewers. Any product that may be evaluated in this article, or claim that may be made by its manufacturer, is not guaranteed or endorsed by the publisher.
